# Two channel passive visualization of a nitinol guidewire with iron markers

**DOI:** 10.1186/1532-429X-17-S1-P236

**Published:** 2015-02-03

**Authors:** Adrienne E Campbell-Washburn, Toby Rogers, Burcu Basar, Merdim Sonmez, Ozgur Kocaturk, Robert J Lederman, Michael Hansen, Anthony Z Faranesh

**Affiliations:** Division of Intramural Research, Cardiovascular and Pulmonary Branch, National Heart Lung and Blood Institute, National Institutes of Health, Bethesda, MD USA; Institute of Biomedical Engineering, Bogazici University, Istanbul, Turkey

## Background

MRI-guidance for cardiovascular catheterization is appealing to reduce ionizing radiation exposure and to enable novel procedures. "Active" guidewire-antennas for MRI-guided procedures are often designed such that the tip and shaft have distinct signals [1] to improve navigation and to make it obvious when the tip moves out of plane. Here we present a method to isolate the signal from iron markers, and produce a two channel color overlay for visualizing the shaft and tip of a nitinol guidewire.

## Methods

Three iron-oxide markers were added to a commercially available 0.035" nitinol guidewire (Nitrex, Covidien, Plymouth, MN). Gradient echo spiral imaging (8 interleaves, TE/TR=0.86/10ms, flip angle = 10°) was performed on a 1.5T MRI scanner (Aera, Siemens, Erlangen, Germany). Image processing was performed in MATLAB (R2013a, Mathworks, Natick, MA).

### Channel 1 - Iron markers

Off-resonance spins, such as those created by the iron markers, cause blurring in spiral images [2]. Images were reconstructed at two frequencies, on-resonance and 200Hz off-resonance. A subtraction of the two image reconstructions [(on-off)/on] generated a characteristic dark-bright-dark pattern from the iron markers, which was detected by a specifically designed convolution kernel.

### Channel 2 - Nitinol guidewire

Through-slice dephasing [3] was applied to alternating frames to generate a positive contrast image, where the nitinol guidewire appears bright with background signal suppressed, from which the guidewire signal was isolated.

## Results

**Figure 1:**
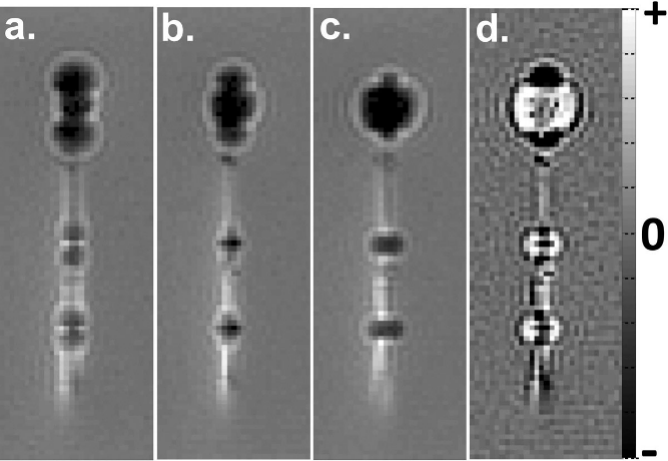
Phantom images of the nitinol guidewire with three iron-oxide markers (one large, two small) demonstrating reconstruction at -200Hz off-resonance (a), on-resonance (b) and +200Hz off-resonance (c). Image subtraction (d) creates a characteristic dark-bright-dark pattern from the iron markers.

**Figure 2:**
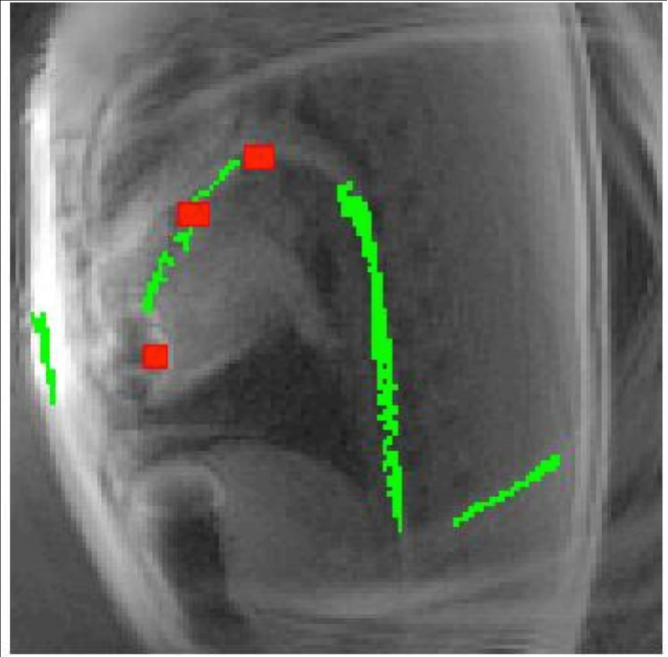
Two channel passive visualization using isolated signal from the iron markers (red) and nitinol guidewire (green).

Spiral images were generated at 80ms/frame. Phantom images show the iron marker signal changing with reconstruction frequency (Figure 1). In vivo, the guidewire was inserted transfemorally to the left ventricle of one pig and imaged. The iron marker signal and nitinol guidewire signal were overlaid on the anatomical image in different colors (Figure 2).

## Conclusions

Here, we have presented a proof-of-concept experiment demonstrating two channel color overlay of a passive nitinol guidewire with iron markers. A new method utilizing off-resonance reconstruction of spiral images was applied to isolate the iron markers and depict them with a unique imaging signature to enhance usability. Future work will optimize the method to ensure robust detection of the marker signal.

## Funding

This work was supported by the NHLBI DIR (Z01-HL006039-01, Z01-HL005062-08).
